# Thiamazol-induzierte Arthritis

**DOI:** 10.1007/s00393-020-00921-0

**Published:** 2020-11-16

**Authors:** Antonia Brinkman, Udo Schneider, Frank Buttgereit, Gerd Burmester, Martin Krusche

**Affiliations:** Medizinische Klinik mit Schwerpunkt Rheumatologie und Klinische Immunologie, Charité Universitätsmedizin, Charitéplatz 1, 10117 Berlin, Deutschland

**Keywords:** Thiamazol, Antithyroidale Medikation, Arthritis, Antithyroid arthritis syndrome, Fieber, Thiamazole, Antithyroid medication, Arthritis, Antithyroid-induced arthritis syndrome, Fever

## Abstract

Wir berichten über den Fall eines 42-jährigen Patienten mit akuter asymmetrischer Polyarthritis der großen und mittelgroßen Gelenke sowie Fieber und erhöhten serologischen Entzündungszeichen. Die Symptomatik begann kurz nach Beginn einer Thiamazol-Therapie bei neu diagnostiziertem Morbus Basedow. Eine durch Thionamide ausgelöste Arthritis wird auch als „antithyroid arthritis syndrome“ (AAS) bezeichnet und ist eine seltene unerwünschte medikamentöse Nebenwirkung. Klinisch kann sich das Krankheitsbild mit Myalgien, Arthralgien, Fieber, Hautausschlag und Polyarthritis präsentieren. Bei Verdacht auf ein AAS sollte die Thionamid-Medikation in Rücksprache mit dem Endokrinologen nach Möglichkeit zeitnah abgesetzt oder umgestellt werden. In einigen Fällen ist eine antiinflammatorische Therapie mit NSAR oder Glukokortikoiden zur Symptomkontrolle nötig.

## Abstract (en)

We report the case of a 42-year-old male patient with acute onset of asymmetrical polyarthritis of the medium and large joints as well as fever and elevated serological inflammation markers. The symptoms began shortly after initiation of thiamazole treatment for newly diagnosed Graves’ disease. Antithyroid arthritis syndrome (AAS) is a rare but serious adverse side effect of antithyroid treatment with thioamides such as thiamazole. Clinically, AAS may present with myalgia, arthralgia, fever, exanthema and polyarthritis. In the case of suspected AAS, when possible the thionamide medication should be rapidly discontinued or modified in consultation with the endocrinologist. In some cases anti-inflammatory therapy with NSAID or corticosteroids may be required for symptom control.

## Fallbericht

Ein 42-jähriger Patient stellte sich notfallmäßig aufgrund von seit 6 Tagen bestehender diffuser, immobilisierender Polyarthritis großer und mittelgroßer Gelenke sowie Fieber bis 38,4 °C stationär vor.

## Vorgeschichte

Fünf Wochen vorher hatte sich der Patient erstmalig anlässlich einer neu aufgetretenen arteriellen Hypertonie mit Tachykardie, Kopfschmerzen, Tremor und ungewolltem Gewichtsverlust von 5 kg (in 1 Monat) ambulant internistisch vorgestellt. Andere Vorerkrankungen oder Zeichen einer endokrinen Orbitopathie lagen nicht vor.

**Figure 1:**
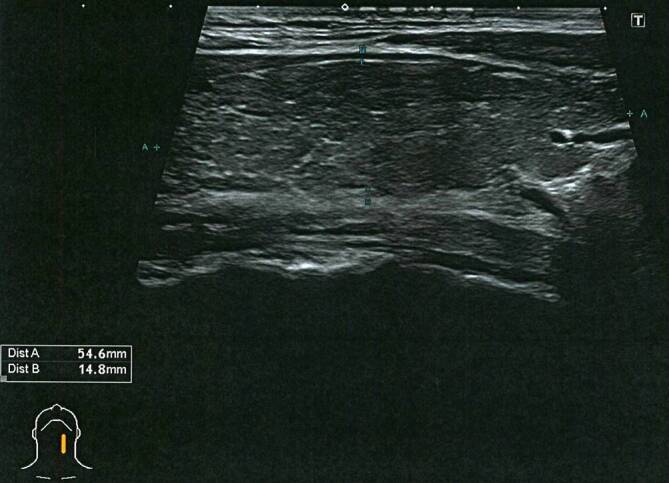

Labordiagnostisch fielen ambulant bereits stark erhöhte Schilddrüsenhormone fT3 von 22,7 pmol/l und fT4 von 55,8 pmol/l (normal fT3 2,8–6,5 pmol/l; fT4 7–21,1 pmol/l) bei einem supprimierten TSH-Wert von <0,005 IU/l (normal 0,3–4,3 IU/l) auf. Gleichzeitig fanden sich erhöhte Thyreoperoxidase(TPO)-Antikörper (196,7 IU/ml) und erhöhte TSH-Rezeptor-Autoantikörper (TRAK) (4,98 IU/l). Die weitere Labordiagnostik erwies sich als unauffällig, insbesondere das C‑reaktive Protein (CRP) lag mit 0,8 mg/l im Normbereich von <5 mg/l. In der Sonographie stellte sich die Schilddrüse beidseits vergrößert, diffus-echoarm, inhomogen und stark perfundiert dar (Abb. 1 und 2). In Zusammenschau der erhöhten Schilddrüsenwerte, korrespondierenden Autoantikörpern und des sonographischen Befundes wurde ambulant die Diagnose eines Morbus Basedow gestellt und eine Therapie mit Thiamazol 20 mg/Tag sowie Metoprolol 100 mg/Tag eingeleitet.

**Figure 2:**
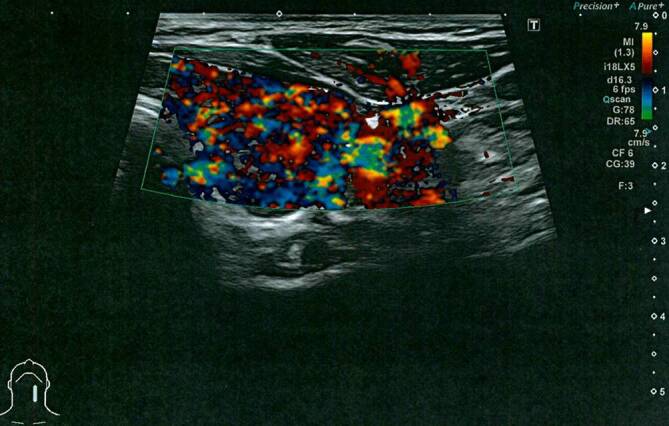

Bei persistierend erhöhten fT3- und fT4-Werten sowie anhaltender Tachykardie (HF 112/min) erfolgte eine schrittweise Erhöhung der Thiamazol-Dosis auf 40 mg/Tag sowie die Umstellung von Metoprolol auf Propanolol (30 mg/Tag).

## Klinischer Verlauf

Zum Zeitpunkt der notfallmäßigen Erstvorstellung in unserer Klinik klagte der Patient über akut erstmalig aufgetretene Arthralgien der folgenden Gelenke: rechtes Schulter‑, Daumensattel- und Kniegelenk sowie linkes Hand- und oberes Sprunggelenk. In der klinischen Untersuchung zeigten sich diese Gelenke druckschmerzhaft, und insbesondere das rechte Kniegelenk war überwärmt und druckschmerzhaft gerötet. Zu diesem Zeitpunkt stand der Patient unter einer Thiamazol-Tagesdosis von 30 mg. Ambulant eingenommene Analgetika (Ibuprofen und Tramadol) hatten nicht zu einer suffizienten Schmerzlinderung geführt. Die Reise- und Berufsanamnese des Patienten war unauffällig.

## Diagnostik

Labordiagnostisch zeigten sich bei Aufnahme bis auf ein erhöhtes CRP von 60,6 mg/l und eine beschleunigte BSG von 60 mm/h bei nach wie vor erhöhten Schilddrüsenhormonen T3 (2,58 μg/l) und fT4 (29,9 ng/l) keine Auffälligkeiten (normales Blutbild; unauffälliges Procalcitonin, Kreatinin und Harnsäure; kein Komplementverbrauch; RF, ACPA, ANA, ANCA, ACE, HLA-B27 negativ). Die infektiologische Testung auf Borrelien, Hepatitis B und C, Gonokokken, Mykoplasmen und Chlamydien war ebenfalls unauffällig.

**Figure 3:**
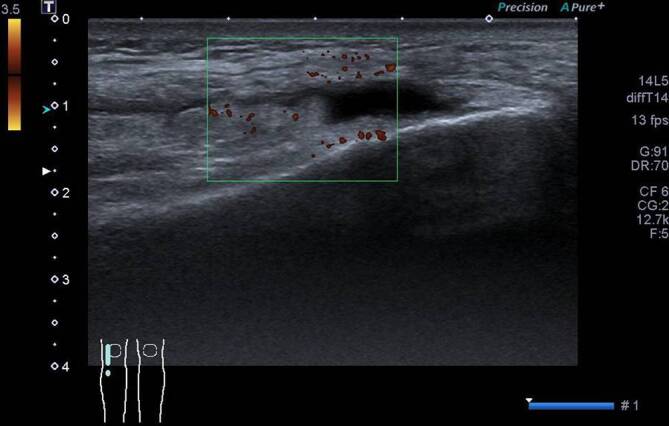

Radiologisch zeigte sich im Röntgenbild der Hände und Füße ein Normalbefund. In der Arthrosonographie sah man einen Kniegelenkerguss mit Synovialitis links (Abb. 3) sowie eine diskrete Synovialitis des rechten Handgelenks und eine OSG-Arthritis links. Das Kniegelenkpunktat zeigte 5142 Leukozyten/µl (Norm <500/µl), davon 4310 Polymorphkernige/µl (Norm <250/µl), ohne Kristall- oder mikrobiologischen Erregernachweis.

## Therapie und Verlauf

In Zusammenschau der Befunde (unauffällige Autoimmunserologie, kein Erreger- oder Kristallnachweis) und unter Berücksichtigung des zeitlichen Zusammenhangs zwischen Beginn der thyreostatischen Therapie und Auftreten der Symptome wurde die Diagnose einer Thiamazol-induzierten Arthritis gestellt.

Initial wurde eine NSAR-Therapie mit Celecoxib (200 mg 2‑mal täglich) eingeleitet, unter der die Schmerzen des Patienten rückläufig waren. Bei jedoch weiterhin steigenden Entzündungsparametern (CRP 113,2 mg/l) und persistierendem Fieber erfolgte eine Prednisolon-Therapie mit 40 mg/Tag (=0,5 mg/kgKG) über 3 Tage. Nach Rücksprache mit der Endokrinologie wurde das Thiamazol abgesetzt und die thyreostatische Therapie auf Propylthiouracil 100 mg 2‑mal täglich umgestellt. Hierunter normalisierten sich die laborchemischen Entzündungszeichen, und die klinischen Beschwerden sistierten innerhalb von 2 Wochen. Das Prednisolon konnte rasch in 1 Monat ausgeschlichen werden, und der Patient war anhaltend beschwerdefrei.

## Diskussion

Der Morbus Basedow mit Nachweis von TSH-Rezeptor-Autoantikörpern (TRAK) stellt in Deutschland mit einer Prävalenz von 20–50/100.000 pro Jahr die zweithäufigste Ursache einer Hyperthyreose dar [1]. Die Erkrankung betrifft Frauen etwa 5‑mal so häufig wie Männer und kann sich mit der klassischen Merseburg-Trias von Struma, endokriner Orbitopathie und Tachykardie präsentieren [1, 2]. In seltenen Fällen sind auch Arthralgien in der Literatur beschrieben [3]. Weiterhin besteht bei vorliegender autoimmuner Schilddrüsenerkrankung ein deutlich erhöhtes Risiko für das Auftreten weiterer Autoimmunerkrankungen [4].

Therapie der ersten Wahl sind Betablocker zur Symptomkontrolle sowie Thyreostatika. Thionamide stellen hier v. a. bei ausgeprägter Symptomatik durch ihren schnellen Wirkeintritt die Therapie der Wahl dar [5]. Bei Therapieversagen kann zur Zweitlinientherapie auch eine Radiojodtherapie eingesetzt oder die Schilddrüse operativ entfernt werden [5, 6].

Das in Deutschland primär verschriebene Thionamid Thiamazol zeichnet sich durch eine längere Wirkdauer (24 h), das schnellere Erreichen einer Euthyreose sowie durch ein günstigeres Sicherheitsprofil im Vergleich zu Propylthiouracil aus [7, 8]. Dennoch reichen die unerwünschten Arzneimittelwirkungen (UAW) von milden Nebenwirkungen wie Juckreiz, Hautausschlägen, Arthralgien, Myalgien, Fieber und Übelkeit bis zu seltenen, aber potenziell schwer verlaufenden Nebenwirkungen wie Agranulozytose, Hepatotoxizität oder Pankreatitis [6, 9].

Laut Literatur kann es in ca. 1,6 % der Fälle zu einer Polyarthritis als Folge einer Thionamid-Medikation kommen [10, 11]. Im Englischen auch als „antithyroid arthritis syndrome“ (AAS) bezeichnet tritt diese UAW meist innerhalb der ersten 2 Monate nach Therapiebeginn auf und betrifft, der Epidemiologie des Morbus Basedow folgend, hauptsächlich Frauen [10, 12]. Neben der Arthritis, die alle peripheren Gelenke betreffen kann, können im Rahmen des AAS ebenfalls Arthralgien, Myalgien, Fieber und Hautveränderungen (Exantheme, Pruritus) auftreten [10, 12, 13]. Laborchemisch zeigen sich eine CRP-Erhöhung sowie eine beschleunigte Blutsenkungsgeschwindigkeit ohne Nachweis von spezifischen Autoantikörpern.

Als seltene medikamentös toxische Nebenwirkung können Thionamide neben einem AAS auch eine ANCA-Vaskulitis oder ein Lupus-like-Syndrom induzieren [6, 9, 14].

Die Pathophysiologie des AAS ist noch nicht abschließend geklärt. Aktuelle Theorien sind:

1. Thionamide und ihre Metabolite werden von neutrophilen Granulozyten aufgenommen, wo sie an die Myeloperoxidase binden und diese sowie andere Proteine wie Lactoferrin, Elastase etc. in Immunogene umwandeln.
2. Individuen mit Defizit in der zellulären Kupferbindungskapazität weisen vermehrt freies Kupfer auf, welches an Thionamide binden kann. Der entstehende Komplex beeinflusst den Glutathionstoffwechsel, was zu Interleukinfreisetzung und zu einer Synovialitis führen kann.
3. Die Thiol-Gruppe der Thionamide bindet an Makromoleküle und agiert so mit diesen als Hapten im Sinne der Induktion einer Antikörperproduktion [10, 12, 15].

Bei Verdacht auf ein AAS sollte das auslösende Medikament nach Rücksprache mit der Endokrinologie abgesetzt werden [9, 10, 12, 13]. In der Mehrheit der Fälle ist die Symptomatik danach rückläufig, jedoch kann eine Gabe von NSAR zu einer schnelleren Linderung der Beschwerden beitragen. In schweren Fällen kann auch der Einsatz einer Glukokortikoidtherapie zur Entzündungshemmung sinnvoll sein [12]. Des Weiteren wurde in einer aktuellen Arbeit über die Wirksamkeit von Colchicin bei einer Glukokortikoid-resistenten Patientin berichtet [10]. Eine regelmäßige Kontrolle der Schilddrüsenfunktionsparameter ist notwendig, und die weitere thyreostatische Therapie sollte engmaschig mit der Endokrinologie abgestimmt werden.

In der klinischen Praxis werden Thionamide v. a. temporär in der Therapieinitiation bei Hyperthyreose angewandt. Meist kann die Therapie nach Normalisierung der Schilddrüsenfunktion nach einigen Monaten wieder beendet werden. Es wird vermutet, dass das AAS dadurch vielleicht sogar häufig unter- bzw. fehldiagnostiziert wird, da bei Unkenntnis des Krankheitsbildes bei initialem Symptombeginn ggf. eine seronegative rheumatoide Arthritis diagnostiziert und eine Therapie mit Basistherapeutika eingeleitet wird [16]. Der Therapieerfolg wird dann nicht auf das reguläre Absetzen der thyreostatischen Medikation zurückgeführt, sondern der immunmodulierenden Therapie zugeschrieben, welche dann unnötig lange fortgeführt wird.

## Fazit für die Praxis

Bei erstmalig akut auftretenden Arthralgien, Myalgien, Hautausschlägen oder Polyarthritis mit Fieber unter antithyroidaler Therapie mit Thionamiden, sollte differenzialdiagnostisch auch an ein „antithyroid arthritis syndrome“ gedacht werden. Therapeutisch genügt meist das Absetzen des Thionamids, jedoch kann der Einsatz von NSAR oder Glukokortikoiden notwendig sein. Eine engmaschige Abstimmung bezüglich der thyreostatischen Therapie sollte in Absprache mit der Endokrinologie erfolgen.
